# National Association

**Published:** 1878-06

**Authors:** 


### Editorial.

### NATIONAL ASSOCIATION.

The organization of a National Association, differing, in some features at least, from any existing body, one that shall represent the whole dental profession of the United States, is now contemplated and is in process of formation. The American Dental Convention, organized about twenty-four years, ago accomplished great good for the profession. By it the members of the profession were brought together in much larger numbers than ever before, and so necessarily became more extensively and more intimately acquainted, and from that time the power of association began to be felt as never before in the ranks of the profession, and not only did those who were members of the body from time to time experience this fraternizing and unifying influence, but those with whom they came in contact outside, experienced an elevating influence that was new to them, and for which they could hardly account. And in addition to this, many new thoughts, ideas and modes of practice were brought forward, presented and became common property. Thus practical knowledge was increased and the sphere of usefulness of the dentist enlarged. The things here mentioned as accomplished, constituted but a very small part of the whole.

A decade or more of years having passed, a new organization seemed to be demanded, one that in some respects should occupy a different level from that of the convention; it was necessary that a work the latter had never attempted should be done. With this end in view, in 1859, the American Dental Association was organized; the character and work of this organization are familiar to all; let it suffice here to say that it has accomplished for the profession and for the public incalculable benefit; but the time has arrived when some of its modes of work may be improved, and others perhaps, in the progress of things, have become obsolete or inefficient.

In effecting a new organization, therefore, two prominent features should be kept in view, viz: securing and promoting the utmost harmony and co-operation of the whole profession in this country; and second, arranging and adapting its modes of operation and work to the needs, demands and possibilities of the times.

In the accomplishment of these ends, wisdom, discretion, patience and persevering work will be required. Many suggestions, doubtless, will be made and plans proposed, each of which should be duly considered, its merit appreciated and utilized.

There will be differences of opinion; these should be respected, considered and harmonized.

In reference to the composition of this new organization, it should be only of delegates from state societies, and these delegates should be the best men of the profession in the respective states, representative men, so far as possible; this can only be done by constituting the body of delegates from state societies. An argument for receiving delegates from state societies only, is found in the fact that nearly all the best men of the profession are to be found in the state societies; and it would encourage and stimulate the organization and support of state societies.

It is doubtful whether a large representation would best accomplish the desired object. With the present status and condition of the state societies, one delegate from each ten members would be quite sufficient. One in seven has been suggested, but we are fully impressed that one delegate for every ten would be the better representation. Those sent as delegates and received into full membership, should be regarded as permanent members, till their formal withdrawal or dismissal. There is no occasion for two classes of members; it produces confusion without any compensating benefit.

The working of the body should be regulated by a *clear*, *well defined* and simple constitution and by-laws. The officers should be such as are usually employed in such organizations; and in some respects it would serve the interests of the body better to elect at each alternate meeting. The association should decide as to the qualifications of its members, and not simply receive any one as a member because he is sent as a delegate.

The organization should be such as to govern itself as nearly as possible, or at least with the minimum exercise of official function. The meetings should be annual for a few years at least. Upon this point, however, there is some diversity of opinion.

If the chief object of the association is solid, earnest work for professional development and progress, that would be better attained by holding the meeting uniformly at some central locality.

The plan of dividing the body into sections, is one we regard as of doubtful propriety, for the present at least.

